# Audio-tactile cues from an object’s fall change estimates of one’s body height

**DOI:** 10.1371/journal.pone.0199354

**Published:** 2018-06-27

**Authors:** Ana Tajadura-Jiménez, Ophelia Deroy, Torsten Marquardt, Nadia Bianchi-Berthouze, Tomohisa Asai, Toshitaka Kimura, Norimichi Kitagawa

**Affiliations:** 1 UCL Interaction Centre (UCLIC), University College London, London, United Kingdom; 2 DEI Interactive Systems Group, Computer Science Department, Universidad Carlos III de Madrid, Leganés, Spain; 3 Human and Information Science Laboratory, NTT Communication Science Laboratories, NTT Corporation, Atsugi, Kanagawa, Japan; 4 Centre for the Study of the Senses, School of Advanced Study, University of London, London, United Kingdom; 5 Munich Center for Neuroscience, Ludwig Maximilian University, Munich, Germany; 6 UCL Ear Institute, University College London, London, United Kingdom; 7 BKC Research Organization of Social Sciences, Ritsmeikan University, Shiga, Japan; 8 Yoshika Institute of Psychology, Shimane, Japan; University G d’Annunzio, ITALY

## Abstract

When we drop an object from our hands, we use internal models of both our body height and object-motion to predict when it will hit the floor. What happens if the sensory feedback finally received from the impact conflicts with this prediction? The present study shows that such conflict results in changes in the internal estimates of our body height: When the object people dropped takes longer than expected to hit the floor, they report feeling taller and behave as if their legs were longer. This provides the first evidence of cross-modal recalibration of body-height representations as a function of changes in the distant environment. Crucially, the recalibration results from a mismatch between the predicted and actual outcome of an action, the ball’s release and impact, which are causally-related but separated in space and time. These results suggest that implicit models of object-motion can interact with implicit and explicit models of one’s body height.

## Introduction

People rely on estimates of their body size whenever they reach for objects, avoid obstacles or manipulate tools [1–3]. Although the dimensions of one’s limbs do not vary on a moment-to-moment basis, it is now well established that their mental estimates do: mental body-representations are re-calibrated in accordance with immediate bodily sensory feedback received for instance through the embodiment of tools or of objects that resemble body parts [2,4–6]. Here, we tested whether such calibration can occur also for more distant feedback and predictions: Building on evidence that the brain is able to predict objects’ motion in a gravitational environment, we hypothesized that the feedback received from an object dropped from one’s hand will also lead to a recalibration of one’s internalized body size. To support to this claim, we tested whether one’s body-representation is updated when the predicted and actual impact of an object dropped on the floor do not match. According to the physical laws of object motion, the time lag between these two events depends on how far above the floor the object was released and this, in turn, depends on the hand position and how tall the person is.

Previous research has shown that the brain computes internal models of the physical laws of motion (e.g., [7]) and takes them into account to perform basic actions [8]. People will notably anticipate the kinematics of objects, for instance the duration of an object’s fall to the floor. Once vision [9–11] or other cues [12] inform them that they have released the object, they employ internal models of motion of falling objects to predict the time of impact. Evidence of the use of these object-motion models comes in part from studies on space flights, showing that under microgravity conditions astronauts continue expecting the effect of Earth’s gravity on a dropped object for a period of several days after leaving Earth [9]. When objects drop near individuals, the internal representation of their own body height serves as a reference to estimate the fall duration [13] and they expect timely sensory feedback from the object’s impact. This however opens a question: Given the permanence of the internalized models of motion of falling objects [9] and the flexibility of the estimate of one’s body size [2,4–6], will people update the estimate of their own height if their expectations about impact are contradicted by the environment, or, like in our study, by experimental manipulations?

**Fig 1:**
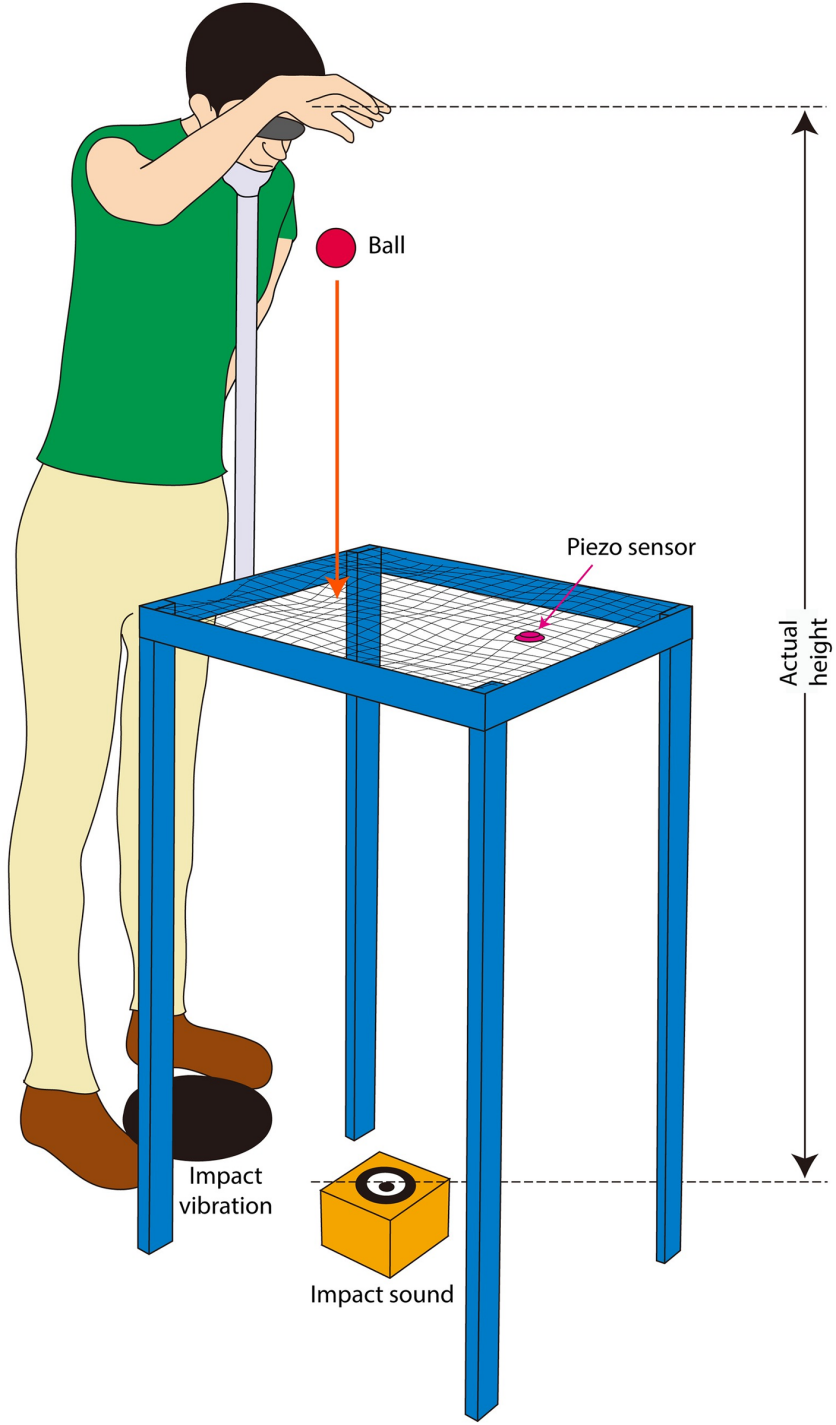
Experimental set-up. Blindfolded participants placed their feet on either side of a mechanical shaker and rested their chin on a chin-rest. They dropped a ball that, unknown to them, fell into a nylon net mounted on a metal frame that silently intercepted the fall of the ball. A piezoelectric transducer attached to the net triggered the audio-tactile feedback simulating the impact of the ball on the floor, after a predetermined delay. A loudspeaker was positioned on the floor beneath the net. White noise was played through two additional loudspeakers placed on the left and right of the participant’s head to mask the weak sound of the ball’s impact onto the net.

We investigated this question in two experiments where participants were handed a ball and were asked to drop it from their full-body height. They received multisensory feedback from the impact of the released object on the floor. This feedback had been experimentally manipulated to be consistent with different dropping heights (Fig 1). More specifically, the experiments consisted of two-minute adaptation phases, in which blindfolded participants were standing and asked to keep the dorsum of their right hand in contact with their forehead. Approximately every 4 seconds, they were handed a plastic ball by the experimenter and they dropped it. Participants were exposed to four different adaptation phases in which the audio-tactile feedback presented near their feet was manipulated to correspond to the ball having fallen from either the participant’s actual height, or from half, or two or three times this height. The maximum of triple simulated height was chosen based on previous reports that the sensory-driven illusion of elongated arm (i.e., a body part) starts breaking when the length of the simulated arm exceeds three times the actual length of the participant’s arm [14,15] and that full body illusions may be induced in which people come to experience ownership of a doll’s body with height 80 cm (approximately half the participants’ actual height) and a giant’s body with height 400 cm (near three times the participant’s actual height) [3].

Experiment 1 aimed to quantify the effects of this multisensory adaptation on participants’ implicit body height representations, while Experiment 2 aimed to quantify the effects on participants’ explicit body height representations. Implicit body-representations are needed to subconsciously act and move, but it is by explicit body-representations that people become aware of their external appearance, in terms of size, shape and the relative position of their limbs [2,3,5,16]. Given the reported dissociations between implicit and explicit body-representation measures [5,17], it was critical to address both types of body-representations.

## Experiment 1: Effects on implicit body height representations

In Experiment 1, we quantified the effects of multisensory adaptation on participants’ implicit body height representation. As these implicit representations are known to guide actions, we measured the pre- and post-performance on a basic action involving body-height representation: Blindfolded participants were asked to take a step backwards, aiming to reach a position behind them that they had memorized prior to each experimental run (similar to [3,18]). Illusory bodily shrinking/expansion is known to influence spatial perception, as it results in people experiencing objects and distances as larger/smaller [3]. If the estimated size of the legs or the entire body had increased by adaptation in response to the increase in simulated height of the ball drop, individuals were expected to take a shorter step, assuming that that step length would suffice to reach the remembered position. On the contrary, if the estimated size of the legs or the entire body had decreased by adaptation in response to the decrease in simulated height of the ball drop, individuals were expected to take a longer step. Given that some previous studies have failed to elicit illusions of body shrinkage as opposed to those illusions of body expansion (e.g., [19–21]), it was important to look separately at the data corresponding to adaptation in response to the increase and to the decrease in simulated height of the ball.

### Materials and methods

#### Participants

Twenty-six participants took part in the experiment (mean age ± SD = 31.27 ± 6.68 years; age range from 22 to 40 years; 11 females and 15 males). Data from the first participant in Experiment 1 were excluded due to errors in the experimental procedure.

In all experiments reported here, participants reported having normal hearing and touch, and were naïve as to the purposes of the study. They were paid for their time and gave their written informed consent prior to their inclusion in the studies. The experiments were conducted in accordance with the ethical standards laid down in the 1964 Declaration of Helsinki and approved by the “Ethics Committee of NTT Communication Science Laboratories”.

#### Apparatus and materials

The experiment was conducted in a sound-attenuated and dimly lit room. The experimental setup used to deliver the audio-tactile feedback is illustrated in Fig 1. This setup was hidden from participants with curtains, which were opened once the participant was blindfolded.

A mechanical bass shaker was placed on the floor, on the top of a squared rubber sheet. A custom-made loudspeaker was positioned on the floor 30 cm in front of the shaker. A plastic ball (diameter 5 cm) was used as the object that participants were to drop from their hand. A metal frame (height 0.75 m) with a thin nylon net mounted on it was placed over the loudspeaker and served to silently intercept the fall of the ball. A piezoelectric transducer attached to the net triggered the audio-tactile feedback after a predetermined delay.

A schema of the connections of the physical components used for the audio-tactile adaptation is shown in S1 Fig. To generate the audio feedback, a “dry” recording of a finger-tap on a cardboard box (125-ms duration, broad spectrum) was loaded onto MAX/MSP software. The different vertical distances (d), travelled by the ball before its impact, were simulated by altering two parameters: the delay of audio-tactile feedback and the intensity of the audio feedback. The delay was calculated according to the Earth’s gravitational constant g (according to the equation: fall duration = sqrt(2xheight/g)), from which the time taken for the ball to get to the nylon net was subtracted. The increased time of sound propagation through air with increased distance was added to the audio feedback. The delay introduced by the system, which mean value is 10.7 +/- 1.8 ms and maximum value measured is 14 ms, was also considered in the calculations. The intensity of the audio feedback decreased with increasing vertical distance according to the inverse square law. Artificial room reverberation was added with constant intensity so that the direct-to-reverberation ratio changed according to the simulated distance of the impact. The actual sound of the ball impact on the net was attenuated by placing a soft fabric on the top of the net and masked by delivering background noise to two additional loudspeakers placed to the left and right of the participant’s head throughout the entire ball-dropping phase (see Procedure). MAX/MSP software was used to control stimulus delivery and record the number of ball drops during the ball-dropping phase.

Participants were exposed to four different simulations of vertical distances, travelled by the ball before its impact. In these four simulations, we manipulated the audio-tactile feedback presented near their feet to correspond to the object having fallen from either the actual height, or from half, two or three times this height. The different simulations were intended, on the one hand, to look at a possible extension of the represented body size with increasing simulated vertical distance travelled by the ball before its impact (actual, double and triple height conditions). On the other hand, they were intended to look at a possible shrinking of the represented body size with decreasing simulated vertical distance travelled by the ball before its impact (actual and half height conditions).

#### Procedure

At the start of the experimental session, the participants’ body height (mean 166.76 ± 7.24 cm; range 152.5–178 cm) and leg length (mean 95.3 ± 5.51 cm; range 87–107 cm) were measured. The former was measured from the top of the head to the floor, and the latter from the hipbone to the floor. These measures were taken to check for possible individual differences in the observed effects due to body/leg height.

The experiment was essentially a sequence of two tasks: “ball-dropping” task (adaptation), and “step” task (measurement). At the start of the experimental session, the participants were instructed in the procedures used in both tasks. For the ball-dropping task, the participants stood placing their feet on either side of the mechanical shaker. They were blindfolded, with their chin placed in a chinrest with their head slightly inclined downwards. The chinrest was used to ensure the ball was always dropped from the same height of approximately 150 cm above their feet. The participants were instructed to keep the dorsum of their right hand in contact with their forehead. For a period of two minutes, the experimenter placed the ball in their right hand approximately every four seconds. The task of the participants was to grab the ball and then release it. The duration of the ball-dropping adaptation phase (two minutes) was chosen according to previous work on a well-known sensory-driven bodily illusion (i.e., the rubber-hand illusion [22]. This duration would allow on average 30 ball drops in each adaptation phase.

In the step task, participants were asked to take a step backwards to get to a remembered position, and the size of their step was measured [3]. The position had been memorized during a practice phase performed prior to each experimental block. In the practice phase, participants were asked to reach for a specific position, located 60 cm behind them, by taking a step backwards with their right foot and receiving visual and verbal feedback on their performance. After repeating this practice task10 times, the participants were then blindfolded and asked to practice the step two more times while receiving verbal feedback on their performance. The experimental block started immediately after the practice phase.

**Fig 2:**
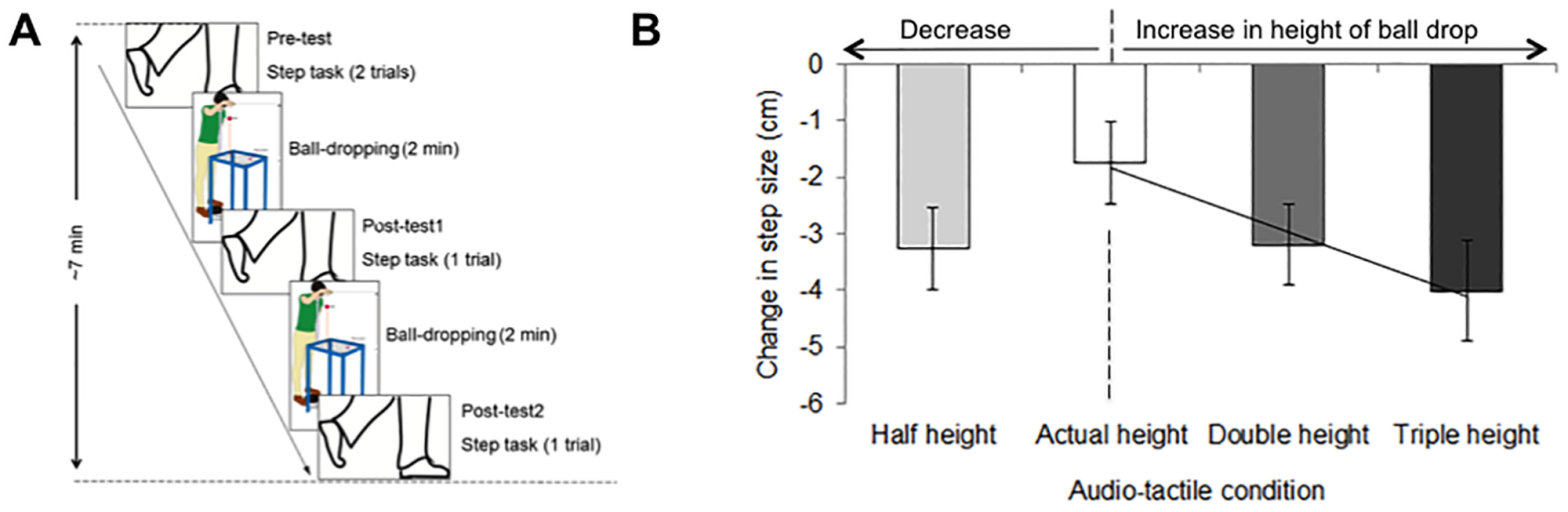
(A) Experimental timeline and (B) results (mean ± SEM) in Experiment 1 on the effect of adaptation to a decrease and an increase in height of the ball drop. Results show that participants’ step size, when aiming to reach a fixed position behind them, was reduced from pre- to post-adaptation. Importantly, a significantly larger decrease was observed when increasing the simulated height of the ball drop. The diagonal line is a linear regression line (y = -1.135 x– 0.7133, R^2^ = 0.9734) and illustrates a decrease in step size with increasing the simulated height of the ball drop during the audio-tactile adaptation. Note that the simulation of the half-height condition failed (see Experiment 3: “Validation of simulations”) and thus the results on this condition do not allow inferring conclusions.

The participants completed four experimental blocks which differed in the simulated height of the ball drop during the adaptation phase: the ball having been dropped from the true height of the participant’s head (actual height condition), from double, triple or half this height (double, triple and half height conditions). Each block had five stages (see Fig 2A): pre-adaptation step task (pre-test, 2 trials), ball-dropping adaptation phase (2 minutes), post-adaptation step task (post-test1, 1 trial), ball-dropping adaptation phase (2 minutes) and post-adaptation step task (post-test2, 1 trial). Participants were not allowed to move their feet during the period between the ball-dropping phase and the step task. The order of the blocks was randomized. Each experimental block lasted approximately 7 minutes. Participants remained blindfolded for the whole duration of the block. They rested between blocks for a period of approximately 10 minutes, during which they did not wear the blindfold. The full experimental procedure including instructions and practice block, one experimental block for each height condition and resting periods, lasted approximately 75 minutes. Note that given the duration of the experiment it was unfeasible to include more repetitions of the experimental trials or blocks; nevertheless, such within-subjects experimental design was preferred to a between-subjects experimental design that would allow more repetitions of one experimental condition to account for the within-subjects variance.

### Results

For each experimental block (actual, double, triple and half height) we averaged the pre-test step size across both repetitions. Preliminary analyses did not show any difference in the pre-test step sizes across the different conditions (all ps > 0.05), thus justifying their choice as baseline. The mean pre-test step sizes (SEM) for all conditions were: actual height: 56.27 (0.73); double height: 56.84 (0.69); triple height: 56.51 (0.81); and half height: 56.21 (0.70) cm. We then calculated for each condition relative step sizes compared to baseline, by subtracting the post-test1 and post-test2 sizes from their respective pre-test baseline. Differences across conditions in the change in step size from pre- to post-test would therefore provide evidence of an effect of the audio-tactile adaptation phase on the represented body/leg height.

First, we submitted relative step sizes in a 4x2 ANOVA with the within-subject factors ‘height condition’ (actual, double, triple and half height) and ‘repetition’ (post-test1 and post-test2). The main effect of ‘height condition’ was significant (F(3,72) = 3.38, p = 0.023), as well as the main effect of ‘repetition’ (F(1,24) = 4.75, p = 0.039). The interaction between both factors failed to reach significance (p = 0.13). Overall, the decrease in step size from pre- to post-adaptation was larger in the post-test2 (mean = -3.42, SEM = 0.65 cm) than in the post-test1 (mean = -2.68, SEM = 0.63 cm), but this effect was independent of the ‘height condition’. Given the significant effect of ‘height condition’, and in order to test our hypotheses, we averaged the post-test relative step sizes across both repetitions and continued to look separately at the data corresponding to adaptation in response to the increase and to the decrease in simulated height of the ball.

Next, our analyses focused on the effect of adaptation to multisensory feedback that appears consistent with an increase in height of the ball drop (see Fig 2B for group means and S2 Fig for individual data). This effect was investigated by conducting planned comparisons to the actual height condition between the means of interest. The comparisons between the relative step size of the actual height condition and those of the double and triple height conditions revealed a significant larger decrease in step size from pre- to post-adaptation in the double (t(24) = 2.34, p = 0.028) and the triple (t(24) = 2.76, p = 0.011) height conditions than in the actual height condition. Moreover, planned contrast analysis showed a significant linear trend (F(1,24) = 7.64, p = 0.011, R^2^ = 0.97), by which the step size decreased with increasing simulated height of the ball drop during audio-tactile adaptation. The mean difference in step size (SEM) for all conditions were: actual height: -1.74 (0.73); double height: -3.2 (0.72); and triple height: -4.01 (0.88) cm.

Finally, the effect of adaptation to multisensory feedback that appears consistent with a decrease in height of the ball drop was investigated by a planned comparison between the relative step sizes of the half height and the actual height conditions. This comparison revealed that the decrease in step size was significantly larger in the half than in the actual height condition (t(24) = 2.45, p = 0.022). The mean difference in step sizes (SEM) were: actual height: -1.74 (0.73); and half height: -3.25 (0.71) cm.

Furthermore, correlation analyses between actual body height and leg length with the differences in step size pre- to post-test for all conditions confirmed that the observed effects were not dependent on participants’ actual body height or leg length (none yield significant results; all ps > 0.17).

### Discussion

In Experiment 1, we had formulated the hypothesis that adaptation to an increase in simulated height of the ball drop would result in individuals taking a shorter step in the step task in the double and triple height conditions, as compared to the actual height condition, assuming that that step length would suffice to reach the remembered position. This pattern of results was confirmed, thus providing evidence of changes in the implicit body-representation in response to adaptation to an increase in simulated height of the ball drop (see Fig 2B).

We had formulated a second hypothesis, which is that adaptation to a decrease in simulated height of the ball drop would result in individuals taking a longer step in the step task in the half height condition, as compared to the actual height condition, assuming that a longer step length is needed to reach the remembered position. This pattern of results was not confirmed. Contrary to our prediction, the results showed a larger decrease in step size in the half than in the actual height condition.

Experiment 2 was conducted in order to investigate the effects of the audio-tactile adaptation in the explicit body-representations by which they become aware of their external appearance. Our aim was to investigate whether the increase in represented body length due to the increase in simulated height of the ball drop reached participants’ awareness. We also aimed at getting further insight on the effects of adaptation to a decrease in simulated height of the ball drop, given the non-expected results for this condition in Experiment 1.

## Experiment 2: Effects on explicit body height representations

In Experiment 2, we quantified the effects of multisensory adaptation on participants’ explicit body height representation. Participants were asked to provide explicit estimates of the length of their legs and entire body using the distance between two vertical lines displayed on a screen [1,23], and to fill in a questionnaire that assessed the subjective experience of participants during the ball-dropping task. If changes in body height representation had occurred by adaptation in response to the changes in simulated height of the ball drop, individuals were expected to change their estimates of the length of their legs and entire body, as well as to indicate the change in their responses to the questionnaire.

### Materials and methods

#### Participants

Twenty-six participants took part in this experiment (mean age ± SD = 30.58 ± 6.18 years; age range from 22 to 39 years; 14 females and 12 males).

#### Apparatus

This experiment used the identical apparatus for the ball-dropping adaptation phase used in Experiment 1. In addition, a projector and a projection screen (3.3 m wide, 3 m away from participants) were used for the aperture task described below. In order to perform this task, participants held a button in their left hand. The software package “Presentation” was used to control stimulus delivery and record responses during the aperture task.

#### Procedure

At the start of the experimental session, the participants’ body height (mean 165.23 ± 7.52 cm; range 152–178 cm) and leg length (mean 94.40 ± 5.14 cm; range 87–107 cm) were measured, as in Experiment 1. Then, the participants were instructed as to the procedures used in the experiment. Experiment 2 was essentially a sequence of two tasks: “ball-dropping” task (adaptation), and “aperture” task (measurement). The ball-dropping phase was identical to the one in Experiment 1.

Before and after each ball-dropping phase, perceived entire body height and leg length were assessed with the aperture task, based on procedures used in previous studies [1,23]. In order to perform this task, participants removed the blindfold without removing their chin from the chinrest. They looked at the projection screen through a small visor, which occluded the space outside the region of the projection screen, so that they could not look down at their body or see the nylon net. In four trials, participants were asked to estimate their entire body height (from foot to head top) or their leg length (from foot to hip bone). Every trial started with the word “body length” or “leg length” being projected, so that participants knew which estimate they had to make. Following this, two vertical lines, horizontally aligned, appeared on the screen. To start with, these lines were 2.5 m apart but they progressively came closer to each other by moving horizontally towards the centre of the screen at approximately 3 cm/s. Participants pressed the button when they felt that the horizontal space (the “aperture”) between the two lines was equivalent to their body height or their leg length, respectively. This was repeated while the aperture was initially closed and the two vertical lines progressively became farther apart. There were two aperture “opening” and two aperture “closing” trials, one of each type for the entire body estimates and one of each type for the leg estimates. The presentation order of these trials was randomized. It should be noted that the estimates were done in the direction perpendicular to the participants’ posture, so that they could not use their eye level as a reference for the estimates (see previous studies by Keizer et al. [1] and Linkenauger et al. [23] for similar methods). Before the experiment started, participants completed a full set of the four trials to familiarize themselves with the aperture task.

**Fig 3:**
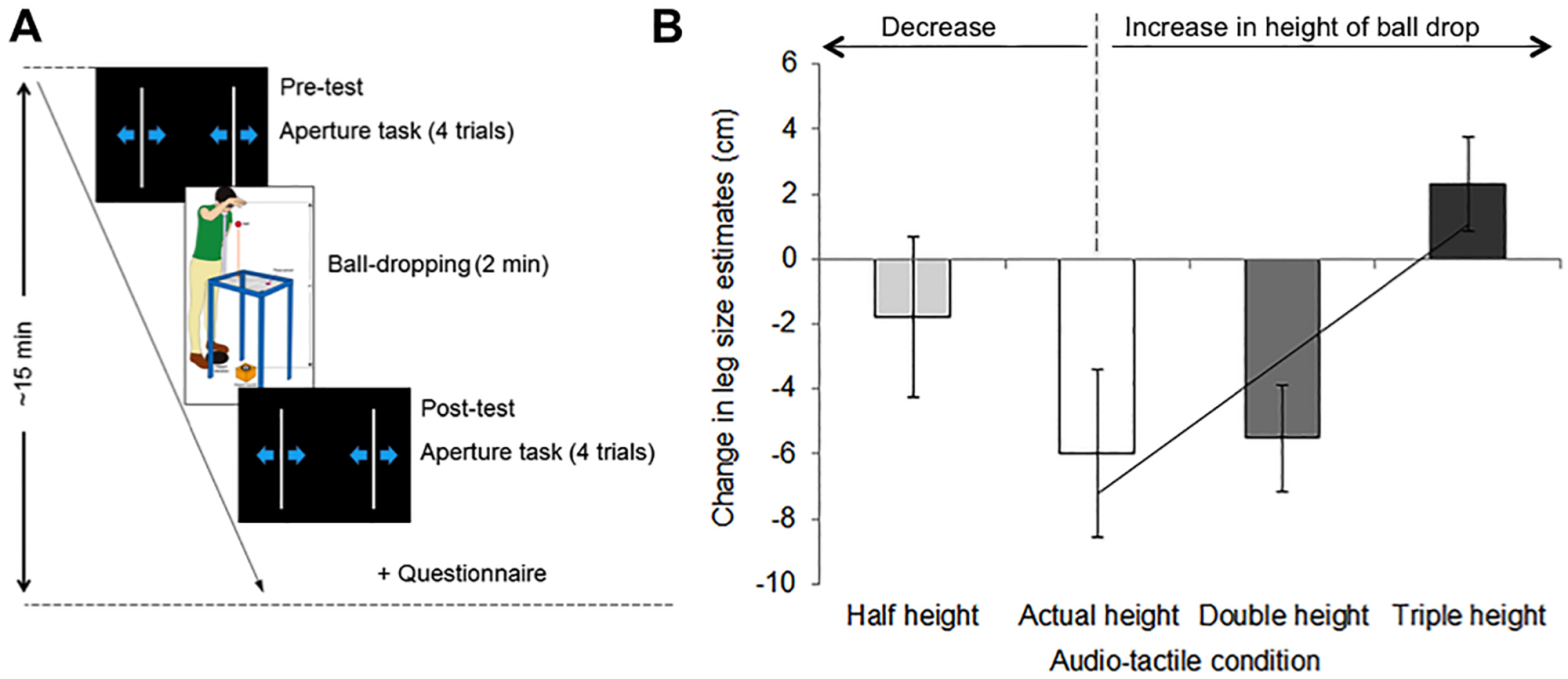
(A) Experimental timeline and (B) behavioural results (mean ± SEM) in Experiment 2 on the effect of adaptation to a decrease and to an increase in height of the ball drop. Results showed that participants’ estimates of their own leg size increased from pre- to post-adaptation in accordance with the increase in simulated height of the ball drop. The diagonal line is a linear regression line (y = 4.1774 x– 11.449, R^2^ = 0.7909) and illustrates a significant increase in leg length estimate with increasing the simulated height of the ball drop during the audio-tactile adaptation. Note that the simulation of the half-height condition failed (see Experiment 3: “Validation of simulations”) and thus the results on this condition do not allow inferring conclusions.

As in Experiment 1, participants completed four experimental blocks in random order (actual, double, triple and half height), each containing four stages (see Fig 3A): pre-adaptation aperture task (pre-test, 4 trials), ball-dropping phase (2 minutes), post-adaptation aperture task (post-test, 4 trials) and questionnaire. Each experimental block lasted approximately 15 minutes. Participants rested between blocks for a period of approximately 20 minutes, during which they did not wear the blindfold.

The questionnaire completed at the end of each block allowed for an assessment of the subjective experience of participants during the ball-dropping task. It contained sixteen statements, adapted from previous studies on bodily illusions [24,25]. Participants rated their level of agreement with the statements using 7-point Likert-type response items, ranging from -3 (strongly disagree) to +3 (strongly agree), with 0 referring to “neither agree, nor disagree” (see statements in S1 Table).

The full experimental procedure including instructions and practice block lasted approximately 135 minutes.

### Results

#### Behavioral results

For each experimental condition, and for both the pre- and post-test, the average height estimate of the “opening” and “closing” aperture task was taken as a measure of entire body and leg height estimation (as in the study by Keizer and colleagues [1]). Given the large individual differences in performance in this task, participants whose average height estimate for at least one of the pre-test estimates differed by more than two standard deviations from the participants’ overall mean were excluded from the analyses, which resulted in the exclusion of four participants. For all statistical tests, the alpha level was set at 0.05, 2-tailed.

Preliminary analyses did not show any difference in the pre-test estimates across the different conditions (all ps > 0.05), thus justifying their choice as baseline. The mean pre-test estimates (SEM) for all conditions were, for the entire body estimates: actual height: 104.49 (6.01); double height: 109.23 (6.41); triple height: 101.88 (5.82); and half height: 107.51 (6.05) cm, and for the leg estimates: actual height: 66.06 (3.50); double height: 67.27 (3.80); triple height: 61.33 (3.51); and half height: 66.46 (4.42) cm. It should be noted that even if the participants provided entire body estimates of approximately 1 meter, which does not correspond to the actual body height of the participants, the variable being quantified is the size of the aperture. The aperture was projected on a screen 3 meters away from the participants. As the participants looked at the projection screen through a small visor that occluded the space outside the region of the projection screen, it might have been difficult for them to know the absolute dimensions of the aperture and every participant may have taken a different reference metric to perform this aperture task. Importantly, our analysis controls for these individual differences in perceived dimensions of the aperture by looking at changes from pre- to post-test. For each condition we calculated relative height estimates compared to baseline, by subtracting the post-test height estimates from their respective pre-test baseline. Differences across conditions in the change in height estimates from pre- to post-test would therefore provide evidence of an effect of the audio-tactile adaptation phase on the represented body/leg height. The mean difference in estimates (SEM) for all conditions were, for the entire body estimates: actual height: -3.05 (2.56); double height: -6.35 (2.67); triple height: -3.56 (2.78) cm; and half height: -7.88 (2.57) cm. For the leg estimates: actual height: -6.03 (2.61); double height: -5.57 (1.65); triple height: 2.32 (1.48) cm; and half height: -1.79 (2.45) cm.

The effect of the simulated distance in the ball-dropping phase was investigated by submitting relative height estimates in two separate ANOVAs, one for each type of ‘body estimate’ (entire body and leg), with the within-subject factor ‘height condition’ (actual, double, triple and half height). The main effect of ‘height condition’ was significant for the leg estimates (F(3,63) = 3.41, p = 0.023), but not for the full body estimates (p = 0.471). The significant effect of ‘height condition’ observed for leg size estimates was further investigated by conducting planned comparisons to the actual height condition. We tested separately the two hypotheses investigating the effect of adaptation to multisensory feedback that appears consistent with an increase in the simulated height of the ball drop and the effect of adaptation to multisensory feedback that appears consistent with a decrease in the simulated height of the ball drop.

First, the effect of adaptation to an increase in the simulated height of the ball drop was investigated by conducting planned comparisons between the relative height estimates in the actual, double and triple height conditions (see Fig 3B for group means and S3 Fig for individual data). There was a significant increase in the leg length estimate from pre- to post-test in the triple height condition, as compared to the actual height condition (t(21) = 2.74, p = 0.012) and the double height condition (t(21) = 3.71, p = 0.001; alpha-level corrected for multiple comparisons was 0.025). Moreover, planned contrast analysis showed a significant linear trend (F(1,21) = 7.53, p = 0.012, R2 = 0.79), by which the estimated length of the legs increased monotonically with increasing impact delay during audio-tactile adaptation.

Second, the effect of adaptation to an increase in the simulated height of the ball drop was investigated by conducting planned comparisons between the relative height estimates in the actual height condition and in the half height condition. This comparison did not reach significance (p = 0.293).

Finally, we confirmed that the observed effects were not dependent on participants’ actual body height or leg length with correlation analyses between actual body/leg height and the differences in estimated body/leg height pre- to post-adaptation for all conditions (none yield significant results; all ps > 0.093).

#### Questionnaire results

The full set of the questionnaire statements, mean responses and results of the statistical tests are presented in S1 Table. Significant non-parametrical Friedman tests, performed between all conditions and for each of the statements, were followed by Wilcoxon signed ranks tests. The post-hoc tests compared the mean response for the actual height condition with the mean responses for the other conditions (with correction for multiple comparisons α = .017). For all conditions, participants agreed that the sound was caused by the ball they dropped (Q1) and that the ball was on the same plane as the sound (Q2). We observed significant differences between the conditions for statement Q2; post-hoc tests showed that participants significantly agreed more with statement Q2 for the for actual height than for the triple height condition, but in all cases participants seemed to agree with this statement (see Experiment 3 for more insight into participants sensations). Participants agreed that their feet were on the same plane as the sound (Q3) for the actual, double and triple height conditions. Note that while levels of agreement for statement Q3 for these three conditions kept generally low, participants clearly disagreed with statement Q3 for the half height condition.

**Fig 4:**
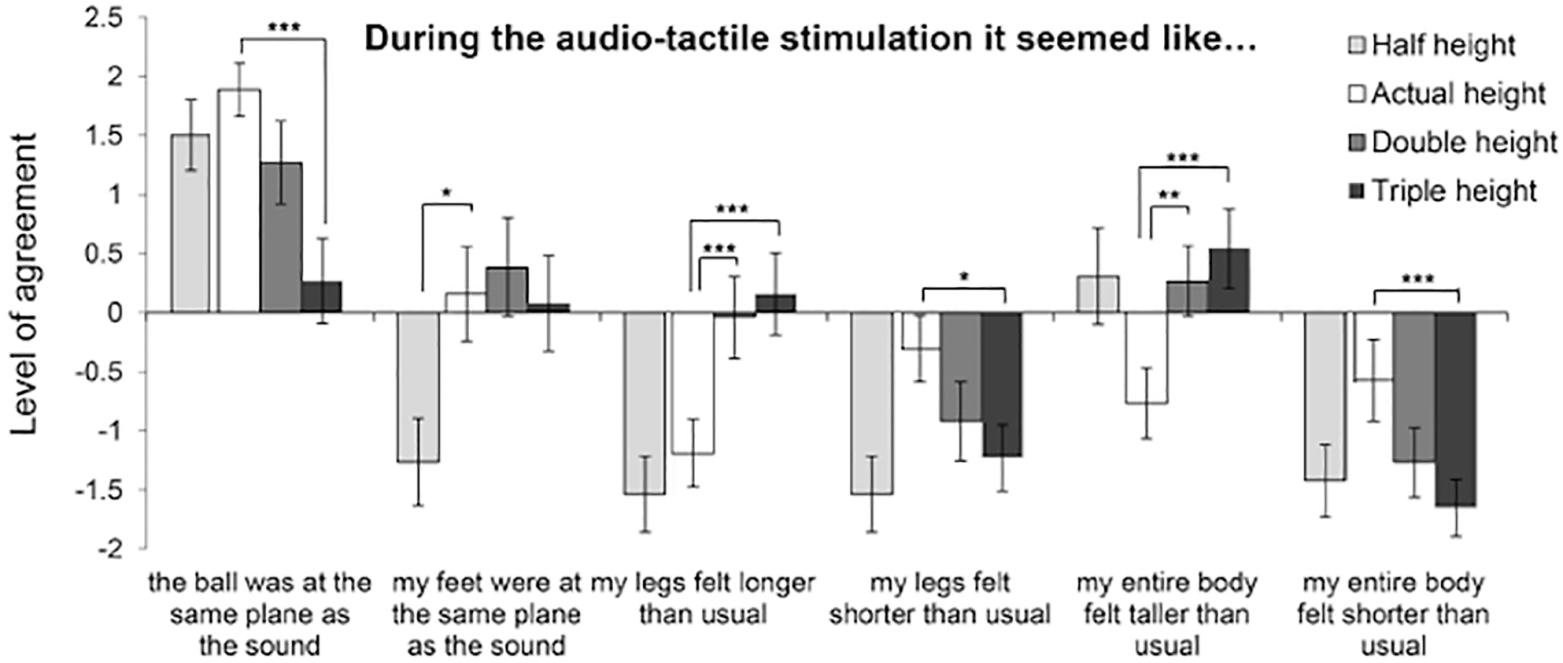
Mean ratings (± SEM) for the questionnaire items related to body size perception that showed significant differences across conditions. Asterisks denote significant differences between means (*** denotes p < .005, ** p < .01 and * p < .05). Note that the simulation of the half-height condition failed (see Experiment 3: “Validation of simulations”) and thus the results on this condition do not allow inferring conclusions.

Additionally, we observed significant differences between the conditions for statements Q4-Q7 (see Fig 4). Paired comparisons revealed that, in the double and triple height conditions, participants agreed more in that their legs felt longer than usual (Q4) and that the entire body felt taller than usual (Q6) than they did in the actual height condition, although levels of agreement with these statements kept generally low. In addition, in the triple height condition participants agreed less in that the legs (Q5) and that the entire body (Q7) felt shorter than usual than they did in the actual height condition. These sensations were however not significantly different between the half and the actual height conditions. These results seem to suggest that the simulated increase in height of the ball drop in the ball-dropping phase resulted in a subjective increase in the perceived length of the legs and the entire body.

### Discussion

In Experiment 2, we had formulated the hypothesis that adaptation to an increase in simulated height of the ball drop would result in individuals providing bigger estimates of leg height and entire body in the double and triple height conditions, as compared to the actual height condition, and that these effects would also reflect in the questionnaire responses. This pattern of results was confirmed in the changes in the leg size estimates (see Fig 3B) and in the questionnaire data (see Fig 4), which proved changes both in the feelings that the legs were longer than usual and that the entire body felt longer than usual. These results provide evidence of changes in the explicit body-representation in response to adaptation to an increase in simulated height of the ball drop. While we do not have evidence to explain the lack of the effects for the entire body estimates in the aperture task, one possibility could be that participants relied on their experience of objects that have their entire body size to provide this estimate, rather than focusing on their current bodily feeling. One participant, for instance, informally reported thinking of the size of his bed when providing this estimate, and said he found it more difficult for the leg size estimate as he did not have a known object to which relate this size.

We had formulated a second hypothesis, which is that adaptation to a decrease in simulated height of the ball drop would result in individuals providing bigger estimates of leg height and entire body in the half height condition, as compared to the actual height condition, and that these effects would also reflect in the questionnaire responses. This pattern of results was not confirmed, as there were no significant changes in provided body estimates between the half and the actual height conditions. Further, participants’ sensations of their legs feeling longer/shorter than usual, or their entire body feeling taller or shorter than usual, were not significantly different between the half and the actual height conditions.

While it is true that there are much fewer reports in previous literature of illusions of body shrinkage than of body expansion (although see for instance [3,26]), self-reports collected in Experiment 2 suggested a failure in the simulation of the half height condition, thus not allowing to infer conclusions from our results on this asymmetry. Self-reports showed that in the half height condition, contrary to what happened for the other three conditions, participants did not feel that their feet were on the same plane as the sound. The binding of the impact sound and the tactile sensation to the feet was critical for a simulation to be successful as it would then let participants associating the stimuli delivered to them (i.e., sound and tactile sensation to the feet) with the ball dropping near their feet. In light of this result, and given the non-expected results for the half height condition in Experiments 1 and 2, we decided to run Experiment 3 (“Validation of simulations”), in order to further check the suitability of the simulations of dropping heights employed in Experiments 1 and 2.

## Experiment 3: Validation of simulations

This “validation of simulations” experiment aimed to further investigate participants’ localization of the stimuli delivered in order to understand whether they appropriately simulated a ball dropping near the feet from different vertical heights, thus allowing to draw conclusions based on the data collected in Experiments 1 and 2.

### Materials and methods

#### Participants

Thirteen participants took part in this experiment (mean age ± SD = 32.62 ± 6.05 years; age range from 26 to 47 years; 7 females and 6 males).

#### Apparatus and materials

This experiment used the identical apparatus for the ball-dropping adaptation phase as was used in Experiments 1 and 2. Five conditions were tested. The conditions differed in their simulated vertical distance travelled by the ball before its impact: the ball having been dropped from the true height of the participant’s head (actual height condition), from double, triple, half or three quarters this height.

#### Procedure

At the start of the experimental session, the participants were instructed in the ball-dropping task. This was performed as in Experiments 1 and 2: the experimenter placed the ball in the right hand of participants, who were asked to grab the ball and then release it. Immediately after each ball-dropping trial, participants were asked to answer two questions: “At which level with respect to your body do you perceive the sound to be?” and “At which level with respect to your body do you perceive the ball has impacted?”. These questions allowed assessing the subjective experience of participants during the ball-dropping task. There were five possible answers for each of the questions: “a) around your chest”, “b) around your waist”, “c) around your knees”, “d) around your feet”, and “e) below your feet”. There was only one experimental block, which included 20 repetitions of each experimental condition. All conditions were intermixed and presented in a random order, with the condition varying from ball-dropping trial to trial.

### Results

**Fig 5:**
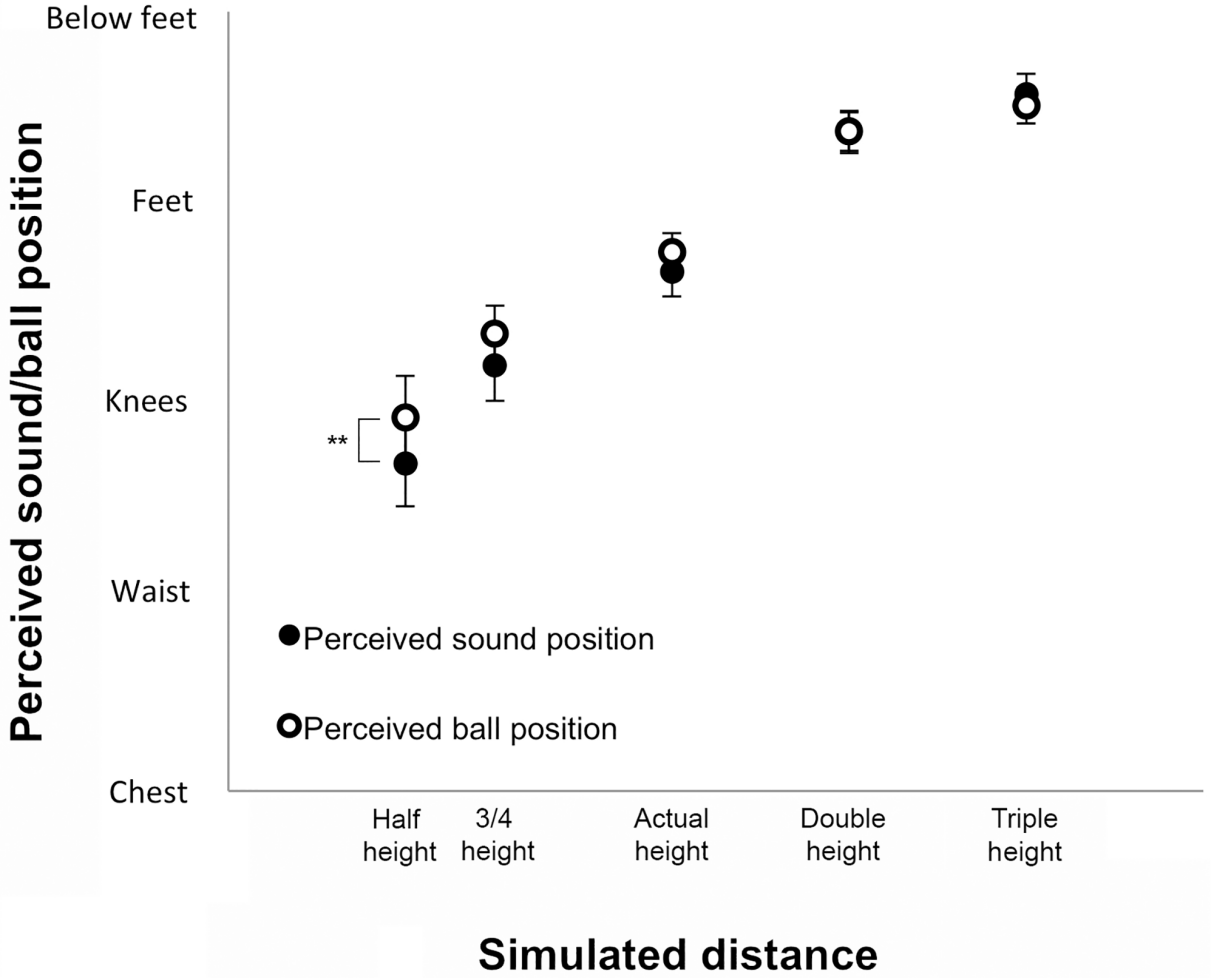
Mean self-reported perceived position (± SEM) for the sound and the ball impact across conditions. Asterisks denote significant differences between means of perceived sound and ball position (** denotes p < 0.01).

The five possible answers to the questionnaire items were coded with numbers from 0 to 4 (i.e., ‘0’ for ‘chest’, ‘1’ for ‘waist’, ‘2’ for ‘knees’, ‘3’ for ‘feet’ and ‘4’ for ‘below feet’). The mean responses and tests for significance for the two questionnaire items are displayed in Fig 5 (see S4 Fig for individual data). An inspection of the data displayed reveals that while for the ‘actual’, ‘double’ and ‘triple height’ conditions participants reported perceiving the sound and the impact of the ball near their feet, this was not the case for the ‘half height’ condition. In this condition, participants reported perceiving the sound and the impact of the ball above their knees.

We conducted non-parametrical Wilcoxon Signed Ranks two-tailed tests on the self-reported data to compare the ratings given for ‘sound location’ and ‘ball impact location’ for each condition against the scores ‘2’ corresponding to ‘knees’, ‘3’ corresponding to ‘feet’, and ‘4’ corresponding to ‘below feet’. Note that with corrections for multiple comparisons the significance level for an alpha set at 0.05, was at p = 0.01. When comparing against the score ‘2’ corresponding to ‘knees’, for self-reported ‘sound location’ we observed significant differences for ‘actual height’ (z = -2.90, p = 0.004), ‘double height’ (z = -3.18, p = 0.001) and ‘triple height’ (z = -3.18, p = 0.001) conditions, but not for ‘half height’ (p = 0.173) nor ‘three quarters height’ (p = 0.294) conditions; similarly, for self-reported ‘ball impact location’ we observed significant differences with the score ‘2’ for ‘actual height’ (z = -3.11, p = 0.002), ‘double height’ (z = -3.18, p = 0.001) and ‘triple height’ (z = -3.18, p = 0.001) conditions, but not for ‘half height’ (p = 0.753) nor ‘three quarters height’ (p = 0.055) conditions. When comparing against the score ‘3’ corresponding to ‘feet’, for self-reported ‘sound location’ we observed significant differences for ‘half height’ (z = -2.18, p = 0.001), ‘three quarters height’ (z = -3.06, p = 0.002), and ‘triple height’ (z = -3.11, p = 0.002) conditions, while there were no significant differences for ‘actual height’ (p = 0.021) and ‘double height’ (p = 0.012) conditions; for self-reported ‘ball impact location’ we observed significant differences with the score ‘3’ for all conditions (‘half height’: z = -3.11, p = 0.002; ‘three quarters height’: z = -2.98, p = 0.003; ‘double height’: z = -2.71, p = 0.007; and ‘triple height’: z = -3.18, p = 0.001) except for ‘actual height’ (p = 0.021). When comparing against the score ‘4’ corresponding to ‘below feet’, for self-reported ‘sound location’ we observed significant differences for all conditions (‘half height’: z = -3.18, p = 0.001; ‘three quarters height’: z = -3.18, p = 0.001; ‘actual height’: z = -3.18, p = 0.001; ‘double height’: z = -3.18, p = 0.001; and ‘triple height’: z = -3.06, p = 0.002); for self-reported ‘ball impact location’ we observed significant differences with the score ‘4’ for all conditions (‘half height’: z = -3.18, p = 0.001; ‘three quarters height’: z = -3.18, p = 0.001; ‘actual height’: z = -3.18, p = 0.001; ‘double height’: z = -3.18, p = 0.001; and ‘triple height’: z = -3.06, p = 0.002). Overall, these results suggest that participants felt the sound location to be around the knees for the ‘half’ and ‘three quarters height’ conditions and around the feet for the ‘actual’ and ‘double height’ conditions, and they were uncertain to whether the sound was on the feet or below the feet for the ‘triple height’ condition as scores for this condition were between 3 and 4 but significantly different from both. Regarding the ball impact location, participants felt it to be around the knees for the ‘half’ and ‘three quarters height’ conditions and around the feet for the ‘actual height’ condition, and they were uncertain to whether the ball impact was on the feet or below the feet for the ‘double’ and ‘triple height’ conditions.

Furthermore, we conducted additional non-parametrical Wilcoxon Signed Ranks two-tailed tests to analyze the differences in perceived ‘sound location’ and ‘ball impact location’ for each condition. Note that with corrections for multiple comparisons the significance level for an alpha set at 0.05, was at p = 0.01. We observed significant differences between self-reported ‘sound location’ and ‘ball impact location’ for the ‘half height’ condition (z = -2.67, p = 0.008). This difference was not significant for the other conditions (‘three quarters height’: p = 0.032; ‘actual height’: p = 0.203; ‘double height’: p = 0.814; and ‘triple height’: p = 0.424).

### Discussion

The four simulations used in Experiments 1 and 2 were validated in a separate experiment, which looked at participants’ perceived location of the auditory feedback and tactile feedback with respect to their body. This experiment showed that participants in the actual height, double height and triple height conditions localized the auditory and tactile feedback approximately at the same location and near their feet, which matched our criteria set to consider the simulations as valid. On the contrary, in the half height condition participants localized sound and tactile feedback at different locations, with both stimuli being localized well above their feet. This may be due to the difficulty to simulate a sound source close to the body by a more distant loudspeaker. These results were in line with the self-reports obtained in Experiment 2, which confirmed that the impact sound and the tactile sensation to the feet were successfully integrated in the actual, double and triple height condition, such that participants felt that the sound was caused by the ball dropping near their feet, but not when trying to simulate a drop of half the actual height (see S1 Table for questionnaire data on the failed half height simulation).

Given that the binding of the impact sound and the tactile sensation to the feet was critical for a successful simulation of a ball drop near participants’ feet, we considered our simulation failed for this half height condition and that results on this condition do not allow inferring conclusions on a sensory-induced illusion of owning a shorter body. We do not think that the null results in this condition were due to technical problems, but rather that they were due to the fact that the simulated shortening was too extreme to be plausible. It might have felt to the subject that the ball was dropped at a table, still giving tactile feedback to the feet via the table legs, which would be a familiar situation. On the contrary, the simulations of the ‘actual height’, ‘double height’ and ‘triple height’ conditions seemed successful in this sense. Though the simulations of the ‘double height’ and ‘triple height’ conditions may have also appeared extreme to participants, they may have been more difficult to relate to a familiar alternative situation. Note that in our pilot studies we had learnt that sound cues (with varying time and intensity) per se were not sufficient to simulate the impact of the ball on the same plane as where the feet were, as our pilot participants reported feeling as if the ball was falling into a hole, or feeling as being at the edge of a cliff. Adding tactile cues to the feet solved this problem, as participants then reported feeling that the ball and their feet were on the same plane as the sound, thus making successful the binding of the sound and tactile sensations to the feet. For this reason, in the remaining of this paper we focus on discussing the results of these three conditions, which allowed us to draw conclusions on a sensory-induced illusion of owning a taller body. In any case, it should be noted that in both Experiments 1 and 2 we checked that ANOVAs on the implicit and explicit data on represented body height when considering the four conditions yielded similar results as those with the three ‘valid’ conditions (i.e., ‘actual height’, ‘double height’ and ‘triple height’).

## General discussion

Our findings provide new evidence describing how body representations adapt to multisensory feedback: If the feedback received from dropping an object appears consistent with a higher dropping height, people report feeling taller and behave as if their legs were longer. That is, adaptation leads to elongation in the explicitly and implicitly represented legs. Implicit changes were evidenced by a shortening of participants’ step size when they were reaching to a remembered position, while explicit changes were evidenced by an increase in the estimated length of the legs, but not of the entire body. In line with previous research, the measured changes of the effect across conditions of the manipulation are different for explicit and implicit measures, highlighting a gap between implicit and explicit body-representations which remains to be investigated (5,17). However, similar tendencies are seen here for both the implicit and explicit tasks, and these tendencies in turn matched participants’ reports of both their legs and their entire body feeling longer than usual. Note that in relation to implicit and explicit body-representations, previous works suggest that unconscious multisensory integration can impact bodily experiences and that the implicit body-representations may come to affect explicit ones [27].

As expected based on the observations of previous related multisensory adaptation studies, our results show a systematic baseline shift from pre- to post-adaptation in all conditions. In two studies that inspired the design of the step task [3,18] a similar systematic shift in a walking task due to multisensory adaptation was observed both in the critical and in the control condition but this shift was smaller for the control than for the critical condition. Similar baseline shifts have also been reported in studies on audiovisual temporal recalibration. That is, exposure to fixed audiovisual time lags for several minutes results in shifts in subjective simultaneity responses in the direction of the exposure lag, indicating a perceptual temporal recalibration of multisensory perception [28,29]. While we cannot fully clarify here whether the baseline change observed in our experiment derives from some sort of perceptual temporal recalibration of multisensory perception [28,29] or other processes, what is critical for our report is that this change in step size from pre- to post-adaptation was significantly smaller in the ‘actual height’ condition (i.e., when the ball-drop delay was undistorted) than in the other conditions.

Furthermore, we observed incomplete recalibrations on body coordinates in response to short periods of multisensory adaptation, but these were also expected based on the observations of previous related multisensory adaptation studies. There is strong evidence that the brain is indeed able to solve the equation for the duration of the fall of an object (fall duration = sqrt(2xheight/g)) as people are accurate in detecting distortions in this duration [12]. While a full reliance on gravitational models and current available sensory information would predict a one-to-one match between changes in simulated height of the drop and changes in body height estimates, our results rather suggest a bias of the prior internalized representation of the body height. We note that the adaptation period was relatively short (2 minutes) compared to participants’ previous experience of their body in interaction with external objects; longer adaptation periods may result in more dramatic changes. Indeed, astronauts take many days of space flights to adapt to the new environment [9]. Hence, although recalibration to multisensory stimuli can sometimes occur very rapidly (see for instance reports of temporal recalibration to audiovisual asynchrony after a single 50-ms trial [30,31] or reports showing that the rubber hand illusion starts after 11 seconds of visuo-tactile stimulation [32]), recalibration of body-representation in response to short periods of multisensory adaptation is often incomplete, as shown in the small proprioceptive drifts measured in the rubber hand illusion (e.g. drifts of less than 3 cm for a rubber hand placed 17.5 cm away from the actual hand; [22,33]) or when simulating out-of-body experiences (e.g., drifts of about 24 cm for a body projected 2 m away from participants [18]). Similarly, the perceptual temporal recalibration of multisensory perception when adapting to fixed audiovisual time lags for several minutes results in shifts in subjective simultaneity responses in the direction of the exposure lag, but these shifts are smaller than the exposure time lag [28,29]. Crucially, a significant decrease in the step size in Experiment 1 and a significant increase in the leg size estimates in Experiment 2 were observed when the simulated height of the drop increased. Note that while in Experiment 1 we observed a larger decrease in step size from pre- to post-adaptation in the post-test2 than in the post-test1, since the effect was independent of the ‘height condition’ the results do not allow conclusions about whether the effect of adaptation increases with exposure time.

This paper adds to the scarce but growing literature on mental representations of the lower limbs and the networks coding the sensory information on these body parts and in the space surrounding them [34–38]. Previous research has suggested that the integration of sensory information on the feet and surrounding space may sometimes differ from the processes described for other body parts such as the hands [36], making relevant the study of the multisensory processes underlying the forming and updating of lower limb representations. Recent studies on this direction have shown, for instance, that people could experience ownership over virtual legs if they observe them being touched simultaneously with touches received in their actual back [37,39], an experience which is closely related to the rubber hand illusion [22,33] or the full-body illusion [18]. Another recent study has measured the size of the multisensory integration area around the feet by measuring speeded responses to vibrations applied to the toes when visual stimuli are approaching the feet, thus extending similar work done for hands, face and trunk [38]. Of relevance to the work presented in this paper, a previous study has shown that depriving participants of auditory and audio-visual information impaired the spatial localization of tactile information on the feet, thus suggesting that spatial localization of the lower limbs depends on multisensory information [38,40]. Our study extends this work by showing that audio-tactile information at the feet, when linked to sensorimotor information distant in space and time by an internal model of object motion, can update the representation of the lower limbs.

Although the neurophysiological mechanisms underlying body and object-motion models are only partly understood [5,7,9,13,16], our results suggest that the two models can interact. This is plausible considering the large overlap of brain regions in the insular cortex and around the temporo-parietal junction, processing visual and vestibular signals to perceive motion of falling objects [13], and integrating somatosensory, visual and auditory signals to form body-representations [16].

While the experimental manipulation varied both the time and sound intensity cues of the impact at the feet, participants might have relied more on timing, which is object and surface independent. Supposing this is the case, the perceived fall duration can be compared to the duration predicted by an internal model of motion of falling objects [9] and the resulting error biases the internalized representation of the body height. These findings significantly add to the current notion that sensory-driven plasticity of internalized body size occurs based only on immediate sensory-motor feedback [5]. In the present case, plasticity occurs based on the mismatch between the predicted and actual outcomes of one’s action, which involves two causally-related events separated in space and time: the ball’s release and the feedback of its impact. Our results therefore provide novel evidence of cross-modal recalibration of models of one’s body height against changes occurring in the distant environment.

## Supporting information

S1 Fig Connections of the physical components used for the audio-tactile adaptation. The signal processing module contained the player and had stored the impact waveform.
(JPG)

S2 Fig Change in step size from pre- to post-adaptation for each participant (N = 25) and two post-test repetitions in Experiment 1. The diagonal lines are linear regression lines (± SEM) and illustrate a decrease in step size with increasing the simulated height of the ball drop during the audio-tactile adaptation. Note that the simulation of the half-height condition failed (see Experiment 3: “Validation of simulations”) and thus the results on this condition do not allow inferring conclusions.
(TIFF)

S3 Fig Change in leg height estimates from pre- to post-adaptation for each participant (N = 22) in Experiment 2. The diagonal line is a linear regression line (± SEM) and illustrates a significant increase in leg length estimate with increasing the simulated height of the ball drop during the audio-tactile adaptation. Note that the simulation of the half-height condition failed (see Experiment 3: “Validation of simulations”) and thus the results on this condition do not allow inferring conclusions.
(TIFF)

S4 Fig Mean self-reported perceived position for the sound and the ball impact across conditions, for each participant (N = 13) and for each of the 20 repetitions (20). The diagonal line is a linear regression line (± SEM).
(TIFF)

S1 Table Mean ratings (SEM) and Friedman tests for each questionnaire item across all conditions, including the failed half height condition. Questionnaire statements Q1-Q16 used 7-point Likert-type response items, ranging from -3 (strongly disagree) to +3 (strongly agree). Significant tests were followed by pairwise comparisons (Wilcoxon Signed Ranks Tests) between the actual height and the double and triple height conditions. Significant comparisons (corrected for multiple comparisons—α = 0.017) are marked in bold font.
(DOCX)

## References

[1] Keizer A, Smeets MAM, Dijkerman HC, Uzunbajakau SA, van Elburg A, Postma A. Too Fat to Fit through the Door: First Evidence for Disturbed Body-Scaled Action in Anorexia Nervosa during Locomotion. PLoS One. 2013;8(5):1–7. 10.1371/journal.pone.0064602 PMC3667140 23734207

[2] Maravita A, Iriki A. Tools for the body (schema). Trends Cogn Sci. 2004;8(2):79–86. doi: 10.1016/j.tics.2003.12.008 15588812 10.1016/j.tics.2003.12.008

[3] van der Hoort B, Guterstam A, Ehrsson HH. Being Barbie: The Size of One’s Own Body Determines the Perceived Size of the World. PLoS One. 2011;6(5):e20195 doi: 10.1371/journal.pone.0020195 21633503 10.1371/journal.pone.0020195 PMC3102093

[4] Botvinick M, Cohen J. Rubber hands “feel” touch that eyes see. Nature. 1998;391(6669):756 doi: 10.1038/35784 9486643 10.1038/35784

[5] Longo MR, Haggard P. What Is It Like to Have a Body? Curr Dir Psychol Sci. 2012;21(2):140–5.

[6] Serino A, Haggard P. Touch and the body. Neurosci Biobehav Rev. 2010;34(2):224–36. doi: 10.1016/j.neubiorev.2009.04.004 19376156 10.1016/j.neubiorev.2009.04.004

[7] Angelaki DE, Shaikh AG, Green AM, Dickman JD. Neurons compute internal models of the physical laws of motion. Nature. 2004;430(6999):560–4. doi: 10.1038/nature02754 15282606 10.1038/nature02754

[8] Gaveau J, Berret B, Angelaki DE, Papaxanthis C. Direction-dependent arm kinematics reveal optimal integration of gravity cues. Marder E, editor. Elife. 2016;5:e16394 doi: 10.7554/eLife.16394 27805566 10.7554/eLife.16394 PMC5117856

[9] McIntyre J, Zago M, Berthoz A, Lacquaniti F. Does the brain model Newton’s laws? Nat Neurosci. 2001;4(7):693–4. doi: 10.1038/89477 11426224 10.1038/89477

[10] Indovina I, Maffei V, Bosco G, Zago M, Macaluso E, Lacquaniti F. Representation of Visual Gravitational Motion in the Human Vestibular Cortex. Science. 2005;308(5720):416 LP–419. 15831760 10.1126/science.1107961

[11] Zago M, McIntyre J, Senot P, Lacquaniti F. Internal models and prediction of visual gravitational motion. Vision Res. 2008;48(14):1532–8. doi: 10.1016/j.visres.2008.04.005 18499213 10.1016/j.visres.2008.04.005

[12] Lacquaniti F, Maioli C. Adaptation to suppression of visual information during catching. J Neurosci. 1989;9(1):149 LP–159. 2913201 10.1523/JNEUROSCI.09-01-00149.1989 PMC6570000

[13] Miller WL, Maffei V, Bosco G, Iosa M, Zago M, Macaluso E, et al Vestibular Nuclei and Cerebellum Put Visual Gravitational Motion in Context. J Neurophysiol. 2008;99(4):1969 LP–1982. 18057110 10.1152/jn.00889.2007

[14] Kilteni K, Normand J-M, Sanchez-Vives M V, Slater M. Extending Body Space in Immersive Virtual Reality: A Very Long Arm Illusion. PLoS One. 2012;7(7):e40867 doi: 10.1371/journal.pone.0040867 22829891 10.1371/journal.pone.0040867 PMC3400672

[15] Tajadura-Jiménez A, Tsakiris M, Marquardt T, Bianchi-Berthouze N. Action sounds update the mental representation of arm dimension: Contributions of kinaesthesia and agency. Front Psychol. 2015;6:1–18. 26074843 10.3389/fpsyg.2015.00689 PMC4448001

[16] Serino A, Alsmith A, Costantini M, Mandrigin A, Tajadura-Jiménez A, Lopez C. Bodily ownership and self-location: Components of bodily self-consciousness. Conscious Cogn. 2013;22(4):1239–52. doi: 10.1016/j.concog.2013.08.013 24025475 10.1016/j.concog.2013.08.013

[17] Longo MR. Implicit and explicit body representations. Eur Psychol. 2015;20(1):6–15.

[18] Lenggenhager B, Tadi T, Metzinger T, Blanke O. Video ergo sum: manipulating bodily self-consciousness. Science. 2007;317(5841):1096–9. doi: 10.1126/science.1143439 17717189 10.1126/science.1143439

[19] Marino BFM, Stucchi N, Nava E, Haggard P, Maravita A. Distorting the visual size of the hand affects hand pre-shaping during grasping. Exp Brain Res. 2010;202(2):499–505. doi: 10.1007/s00221-009-2143-4 20044746 10.1007/s00221-009-2143-4

[20] Tajadura-Jiménez A, Vakali M, Fairhurst MT, Mandrigin A, Bianchi-Berthouze N, Deroy O. Contingent sounds change the mental representation of one’s finger length. Sci Rep. 2017;7(1):5748 doi: 10.1038/s41598-017-05870-4 28720803 10.1038/s41598-017-05870-4 PMC5515978

[21] Haggard P, Jundi S. Rubber hand illusions and size-weight illusions: Self-representation modulates representation of external objects. Perception. 2009;38(12):1796–803. doi: 10.1068/p6399 20192129 10.1068/p6399

[22] Tsakiris M, Tajadura-Jiménez A, Costantini M. Just a heartbeat away from one’s body: Interoceptive sensitivity predicts malleability of body-representations. Proc R Soc B Biol Sci. 2011;278(1717). 10.1098/rspb.2010.2547 PMC3125630 21208964

[23] Linkenauger SA, Witt JK, Bakdash JZ, Stefanucci JK, Proffitt DR. Asymmetrical Body Perception. Psychol Sci. 2009;20(11):1373–80. doi: 10.1111/j.1467-9280.2009.02447.x 19788528 10.1111/j.1467-9280.2009.02447.x PMC2858772

[24] Longo MR, Schüür F, Kammers MPM, Tsakiris M, Haggard P. What is embodiment? A psychometric approach. Cognition. 2008;107(3):978–98. doi: 10.1016/j.cognition.2007.12.004 18262508 10.1016/j.cognition.2007.12.004

[25] Tajadura-Jiménez A, Väljamäe A, Toshima I, Kimura T, Tsakiris M, Kitagawa N. Action sounds recalibrate perceived tactile distance. Curr Biol. 2012;22(13):R516–7. doi: 10.1016/j.cub.2012.04.028 22789996 10.1016/j.cub.2012.04.028

[26] Banakou D, Groten R, Slater M. Illusory ownership of a virtual child body causes overestimation of object sizes and implicit attitude changes. Proc Natl Acad Sci. 2013;110(31):12846–51. doi: 10.1073/pnas.1306779110 23858436 10.1073/pnas.1306779110 PMC3732927

[27] Salomon R, Noel J-P, Łukowska M, Faivre N, Metzinger T, Serino A, et al Unconscious integration of multisensory bodily inputs in the peripersonal space shapes bodily self-consciousness. Cognition. 2017;166:174–83. doi: 10.1016/j.cognition.2017.05.028 28577447 10.1016/j.cognition.2017.05.028

[28] Fujisaki W, Shimojo S, Kashino M, Nishida S. Recalibration of audiovisual simultaneity. Nat Neurosci. 2004;7(7):773–8. doi: 10.1038/nn1268 15195098 10.1038/nn1268

[29] Vroomen J, Keetels M, de Gelder B, Bertelson P. Recalibration of temporal order perception by exposure to audio-visual asynchrony. Cogn Brain Res. 2004;22(1):32–5. 10.1016/j.cogbrainres.2004.07.003 15561498

[30] Van der Burg E, Alais D, Cass J. Rapid Recalibration to Audiovisual Asynchrony. J Neurosci. 2013;33(37):14633 LP–14637. 24027264 10.1523/JNEUROSCI.1182-13.2013 PMC6705173

[31] Simon DM, Noel J-P, Wallace MT. Event Related Potentials Index Rapid Recalibration to Audiovisual Temporal Asynchrony. Front. Integr. Neurosci. 2017; 11:8 doi: 10.3389/fnint.2017.00008 28381993 10.3389/fnint.2017.00008 PMC5360737

[32] Ehrsson HH, Spence C, Passingham RE. That’s my hand! Activity in premotor cortex reflects feeling of ownership of a limb. Science. 2004;305(5685):875–7. doi: 10.1126/science.1097011 15232072 10.1126/science.1097011

[33] Tsakiris M, Haggard P. Experimenting with the acting self. Cogn Neuropsychol. 2005;22(3):387–407. doi: 10.1080/02643290442000158 21038257 10.1080/02643290442000158

[34] Schicke T, Röder B. Spatial remapping of touch: Confusion of perceived stimulus order across hand and foot. Proc Natl Acad Sci. 2006;103(31):11808 LP–11813. 16864789 10.1073/pnas.0601486103 PMC1544251

[35] Schicke T, Bauer F, Röder B. Interactions of different body parts in peripersonal space: how vision of the foot influences tactile perception at the hand. Exp Brain Res. 2009;192(4):703–15. doi: 10.1007/s00221-008-1587-2 18841353 10.1007/s00221-008-1587-2

[36] van Elk M, Forget J, Blanke O. The effect of limb crossing and limb congruency on multisensory integration in peripersonal space for the upper and lower extremities. Conscious Cogn. 2013;22(2):545–55. doi: 10.1016/j.concog.2013.02.006 23579198 10.1016/j.concog.2013.02.006

[37] Pozeg P, Palluel E, Ronchi R, Solcà M, Al-Khodairy A-W, Jordan X, et al Virtual reality improves embodiment and neuropathic pain caused by spinal cord injury. Neurology. 2017;89(18):1894–903. doi: 10.1212/WNL.0000000000004585 28986411 10.1212/WNL.0000000000004585 PMC5664293

[38] Stone KD, Kandula M, Keizer A, Dijkerman HC. Peripersonal space boundaries around the lower limbs. Exp Brain Res. 2018;236(1):161–73. doi: 10.1007/s00221-017-5115-0 29098315 10.1007/s00221-017-5115-0

[39] Pozeg P, Galli G, Blanke O. Those are Your Legs: The Effect of Visuo-Spatial Viewpoint on Visuo-Tactile Integration and Body Ownership. Front Psychol. 2015;6:1749 doi: 10.3389/fpsyg.2015.01749 26635663 10.3389/fpsyg.2015.01749 PMC4646976

[40] Noel J-P, Wallace M. Relative contributions of visual and auditory spatial representations to tactile localization. Neuropsychologia. 2016;82:84–90. doi: 10.1016/j.neuropsychologia.2016.01.005 26768124 10.1016/j.neuropsychologia.2016.01.005 PMC4752883

