# Supplementary figures and images for: Audio-tactile cues from an object’s fall change estimates of one’s body height
Source: PLoS One. 2018 Jun 27;13(6):e0199354. doi: 10.1371/journal.pone.0199354 (PMC6021069; doi:10.1371/journal.pone.0199354)

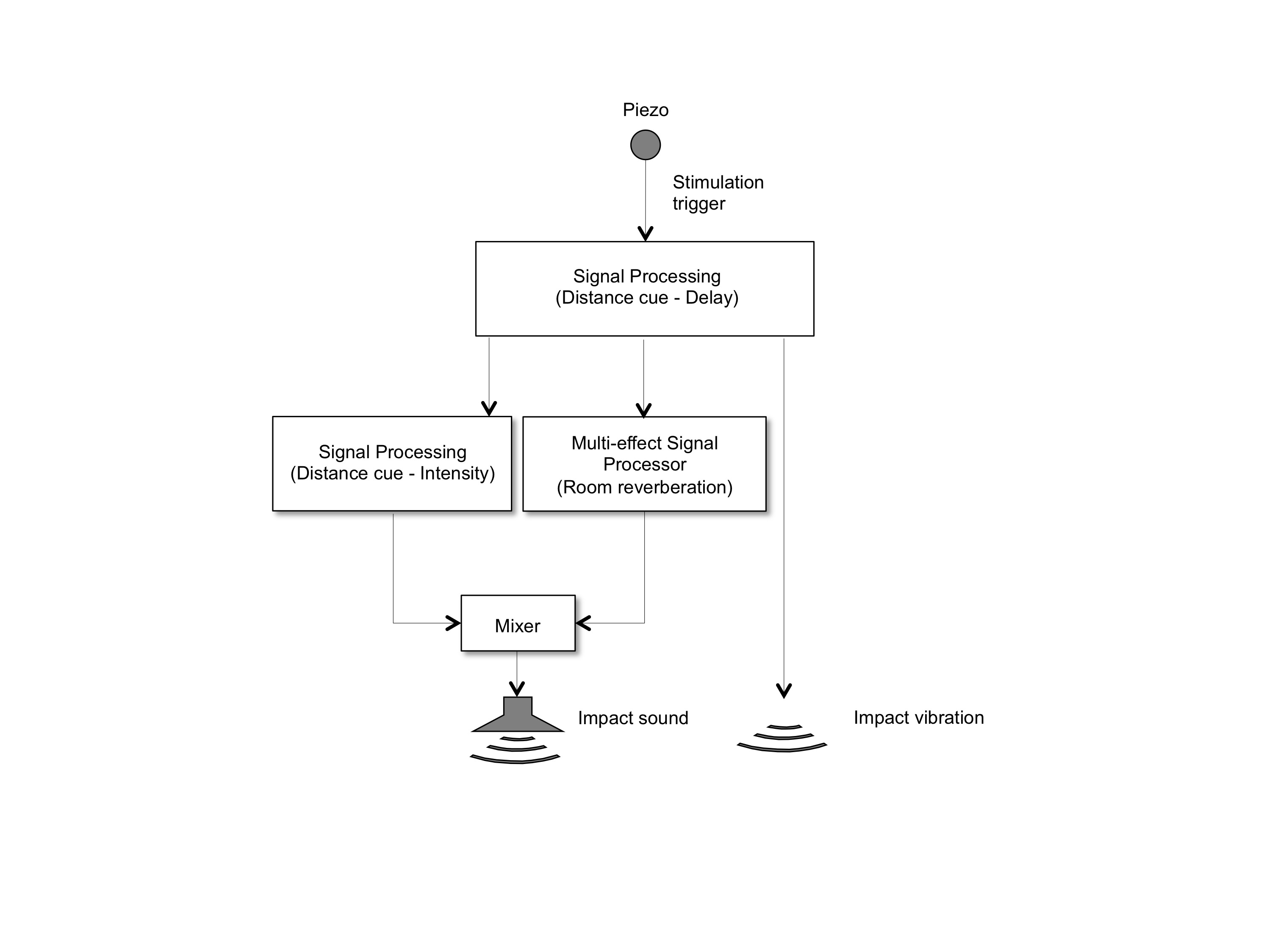

Supplement: S1 Fig — The signal processing module contained the player and had stored the impact waveform. (JPG) [file pone.0199354.s001.jpg]

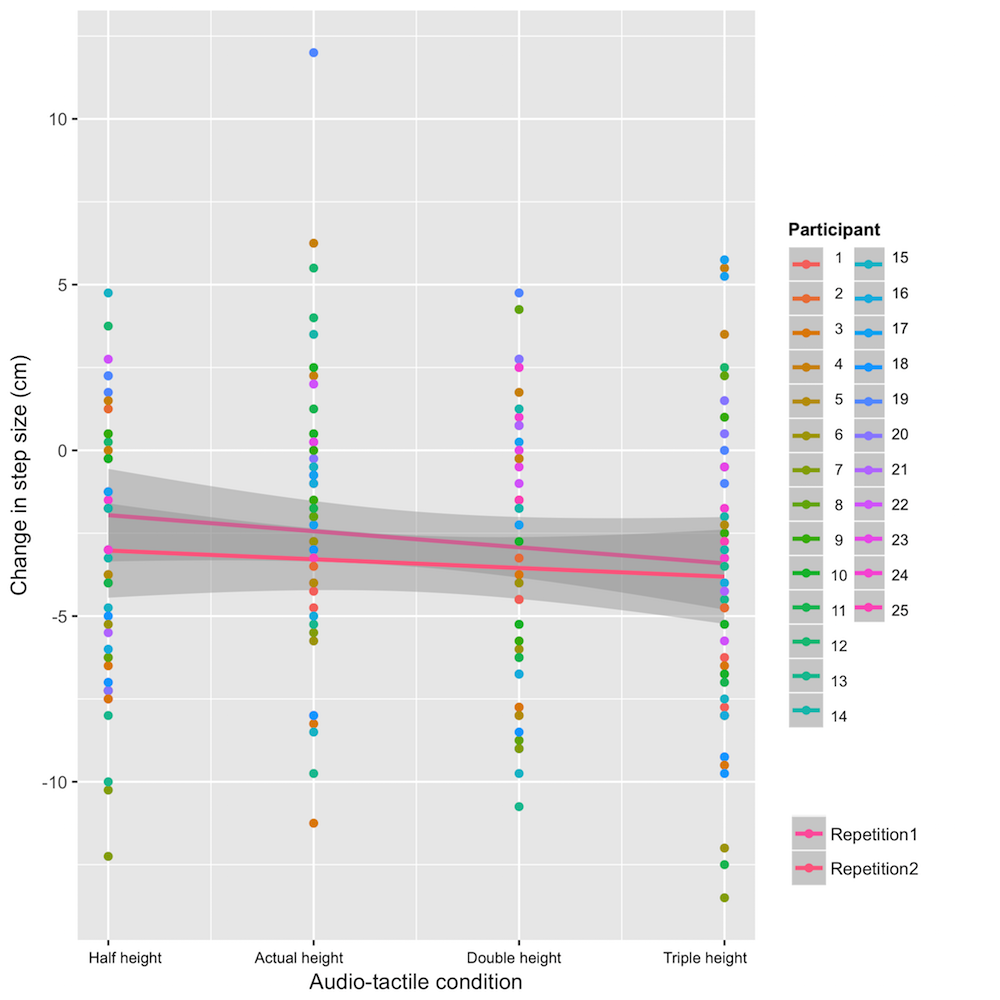

Supplement: S2 Fig — The diagonal lines are linear regression lines (± SEM) and illustrate a decrease in step size with increasing the simulated height of the ball drop during the audio-tactile adaptation. Note that the simulation of the half-height condition failed (see Experiment 3: “Validation of simulations”) and thus the results on this condition do not allow inferring conclusions. (TIFF) [file pone.0199354.s002.tiff]

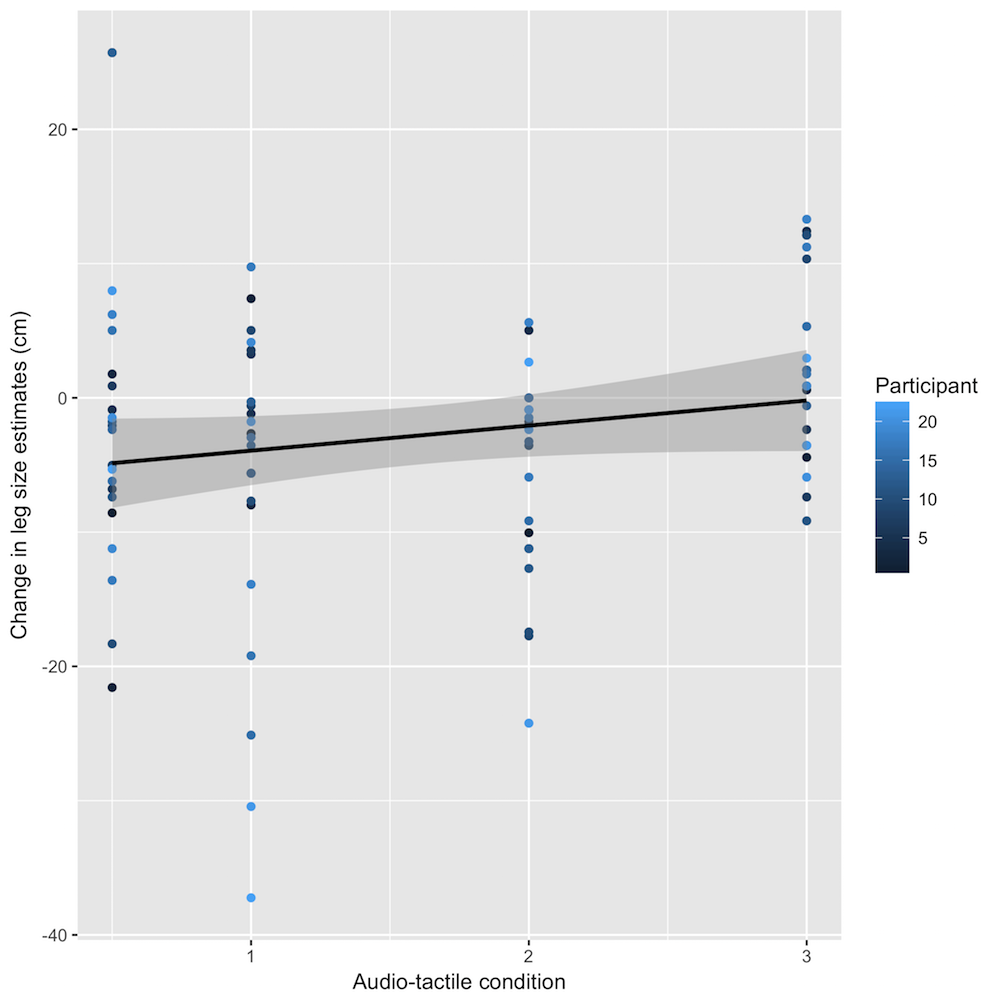

Supplement: S3 Fig — The diagonal line is a linear regression line (± SEM) and illustrates a significant increase in leg length estimate with increasing the simulated height of the ball drop during the audio-tactile adaptation. Note that the simulation of the half-height condition failed (see Experiment 3: “Validation of simulations”) and thus the results on this condition do not allow inferring conclusions. (TIFF) [file pone.0199354.s003.tiff]

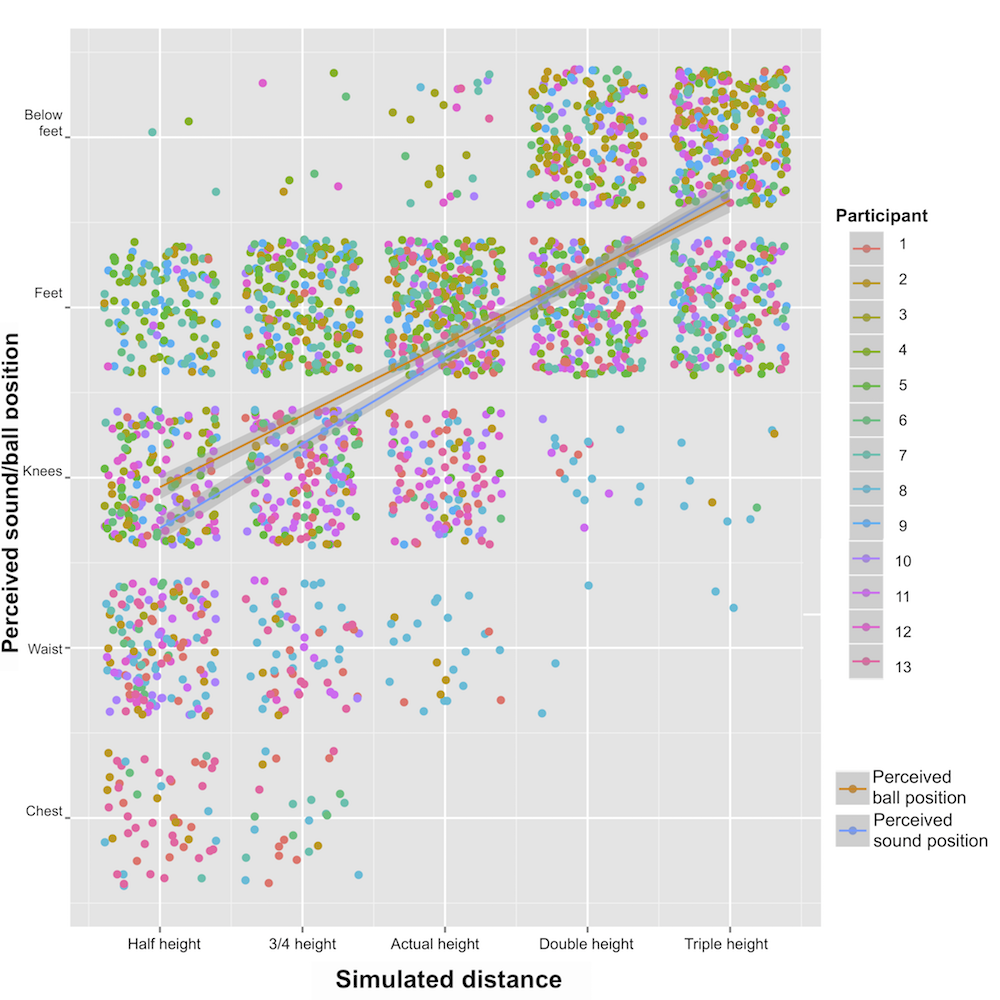

Supplement: S4 Fig — The diagonal line is a linear regression line (± SEM). (TIFF) [file pone.0199354.s004.tiff]
