# Supplementary material for: Audio-tactile cues from an object’s fall change estimates of one’s body height
Source: PLoS One. 2018 Jun 27;13(6):e0199354. doi: 10.1371/journal.pone.0199354 (PMC6021069; doi:10.1371/journal.pone.0199354)
Supplement: S1 Table — Questionnaire statements Q1-Q16 used 7-point Likert-type response items, ranging from -3 (strongly disagree) to +3 (strongly agree). Significant tests were followed by pairwise comparisons (Wilcoxon Signed Ranks Tests) between the actual height and the double and triple height conditions. Significant comparisons (corrected for multiple comparisons—α = 0.017) are marked in bold font. (DOCX) [file pone.0199354.s005.docx]

**Table S1:** Mean ratings (SEM) and Friedman tests for each questionnaire item across all conditions, including the failed half height condition. Questionnaire statements Q1-Q16 used 7-point Likert-type response items, ranging from -3 (strongly disagree) to +3 (strongly agree). Significant tests were followed by pairwise comparisons (Wilcoxon Signed Ranks Tests) between the actual height and the double and triple height conditions. Significant comparisons (corrected for multiple comparisons - α=0.017) are marked in bold font.

| **It seemed like…** | **Mean ratings for all height conditions (SEM)** | **Actual vs. double height** | **Actual vs. triple height** |
| --- | --- | --- | --- |
| Q1:… the sound I heard was caused by the ball I dropped | Actual: 1.65 (0.32)  Double: 1.62 (0.35)  Triple: 0.81 (0.39)  Failed Half: 1.58 (0.35) | z=0.15  p=0.885 | z=1.55  p=0.121 |
|  | χ2 (3) = 2.21, P = 0.53 |  |  |
| Q2:… the ball was at the same plane as the sound | Actual: 1.88 (0.22)  Double: 1.27 (0.35)  Triple: 0.27 (0.36)  Failed Half: 1.50 (0.30) | z=1.61  p=0.107 | **z=3.53**  **p=0.000** |
|  | **χ2 (3) = 20.10, P < 0.001** |  |  |
| Q3:… my feet were at the same plane as the sound | Actual: 0.15 (0.40)  Double: 0.38 (0.42)  Triple: 0.08 (0.40)  Failed Half: -1.27 (0.37) | z=0.48  p=0.634 | z=1.08  p=0.282 |
|  | **χ2 (3) = 12.14, P = 0.007** |  |  |
| Q4:… my legs felt longer than usual | Actual: -1.19 (0.28)  Double: -0.04 (0.35)  Triple: 0.15 (0.35)  Failed Half: -1.54 (0.32) | **z=3.35**  **p=0.001** | **z=3.06**  **p=0.002** |
|  | **χ2 (3) = 25.93, P < 0.001** |  |  |
| Q5:… my legs felt shorter than usual | Actual: -0.31 (0.28)  Double: -0.92 (0.34)  Triple: -1.23 (0.28)  Failed Half: 0.31 (0.41) | - | **z=2.48**  **p=0.013** |
|  | **χ2 (3) = 11.16, P = 0.011** |  |  |
| Q6:… my entire body felt taller than usual | Actual: -0.77 (0.30)  Double: 0.27 (0.30)  Triple: 0.54 (0.34)  Failed Half: -1.42 (0.30) | **z=2.61**  **p=0.009** | **z=2.98**  **p=0.003** |
|  | **χ2 (3) = 22.79, P < 0.001** |  |  |
| Q7:… my entire body felt shorter than usual | Actual: -0.58 (0.35)  Double: -1.27 (0.29)  Triple: -1.65 (0.24)  Failed Half: 0.04 (0.37) | - | **z=2.88**  **p=0.004** |
|  | **χ2 (3) = 24.36, P < 0.001** |  |  |
| **It seemed like…** | **Mean ratings for all height conditions (SEM)** | **Actual vs. double height** | **Actual vs. triple height** |
| Q8:… my body was out of my control | Actual: -0.27 (0.34)  Double: -0.27 (0.27)  Triple: -0.38 (0.30)  Failed Half: -0.15 (0.31) | - | - |
|  | χ2 (3) = 4.51, P = 0.21 |  |  |
| Q9:… I couldn’t remember how long my legs were | Actual: 0.04 (0.33)  Double: 0.34 (0.27)  Triple: 0.27 (0.29)  Failed Half: 0.08 (0.34) | - | - |
|  | χ2 (3) = 0.86, P = 0.83 |  |  |
| Q10:… I couldn’t remember how tall I was | Actual: 0.00 (0.32)  Double: -0.38 (0.27)  Triple: 0.23 (0.30)  Failed Half: -0.31 (0.32) | - | - |
|  | χ2 (3) = 5.61, P = 0.13 |  |  |
| Q11:…I couldn’t really tell where my feet were | Actual: -0.27 (0.27)  Double: -0.27 (0.31)  Triple: -0.77 (0.34)  Failed Half: -0.58 (0.34) | - | - |
|  | χ2 (3) = 2.00, P = 0.57 |  |  |
| Q12:…the experience of my legs was less vivid than normal | Actual: -0.12 (0.31)  Double: 0.08 (0.33)  Triple: -0.04 (0.37)  Failed Half: -0.38 (0.31) | - | - |
|  | χ2 (3) = 1.53, P = 0.68 |  |  |
| Q13:…the experience of my entire body was less vivid than normal | Actual: 0.00 (0.31)  Double: 0.00 (0.28)  Triple: -0.08 (0.33)  Failed Half: -0.42 (0.28) | - | - |
|  | χ2 (3) = 1.84, P = 0.61 |  |  |
| Q14:…the feelings about my body were surprising and unexpected. | Actual: -46 (0.26)  Double: -0.31 (0.29)  Triple: -0.31(0.31)  Failed Half: -0.31 (0.23) | - | - |
|  | χ2 (3) = 1.01, P = 0.80 |  |  |
| Q15:…I found that I had the sensation of pins and needles in my body | Actual: -1.27 (0.40)  Double: -1.23 (0.43)  Triple: -1.46 (0.39)  Failed Half: -1.23 (0.39) | - | - |
|  | χ2 (3) = 1.10, P = 0.78 |  |  |
| Q16:…I found I had the sensation that my legs were numb. | Actual: -1.42 (0.36)  Double: -1.54 (0.37)  Triple: -1.62 (0.36)  Failed Half: -1.42 (0.39) | - | - |
|  | χ2 (3) = 0.18, P = 0.98 |  |  |
